# Actual versus ideal body weight for acute kidney injury diagnosis and classification in critically Ill patients

**DOI:** 10.1186/1471-2369-15-176

**Published:** 2014-11-15

**Authors:** Charat Thongprayoon, Wisit Cheungpasitporn, Abbasali Akhoundi, Adil H Ahmed, Kianoush B Kashani

**Affiliations:** Division of Nephrology and Hypertension, Department of Internal Medicine, Mayo Clinic, 200 First Street SW, Rochester, MN 55905 USA; Division of Pulmonary and Critical Care Medicine, Department of Internal Medicine, Mayo Clinic, Rochester, MN USA; North Central Texas Medical Foundation, Wichita Falls Family Practice Residency Program WFFRP, Wichita Falls, Tx USA

**Keywords:** Acute kidney injury, Actual body weight, Ideal body weight, Urine output

## Abstract

**Background:**

In the current acute kidney injury (AKI) definition, the urine output (UO) criterion does not specify which body weights (BW), i.e. actual (ABW) versus ideal (IBW), should be used to diagnose and stage AKI, leading to heterogeneity across research studies.

**Methods:**

This is a single center, retrospective, observational study conducted at a tertiary referral hospital. All adult patients who were admitted to intensive care units (ICUs) at our institution for a minimum of 6 continuous hours between January and March 2010 and had a urinary catheter for hourly urine output monitoring were eligible for this study. Patients’ AKI stages, based on UO criterion, were assessed by calculating each milliliter of urine per kilogram per hour, using ABW versus IBW.

**Results:**

A total of 493 ICU patients were included in the analysis. The median ABW and IBW were 82 (IQR 68-96) and 70 (IQR 60-77) kg, respectively. Using the IBW criterion, 154 patients (31.2%) were diagnosed with AKI, while 204 (41.4%) were diagnosed using the ABW measurement (*P*-value < .01). Patients who had AKI regardless of BW type had an adjusted odds ratio of 1.76 (95% CI 1.05-2.95) for 90-day mortality, whereas patients who had AKI according to ABW but not IBW had no significant increase in the risk of 90-day mortality, adjusted OR 0.76; (95% CI 0.25-1.91), compared to patients who had no AKI.

**Conclusions:**

Using ABW to diagnose and stage AKI by UO criterion is more sensitive and less specific than IBW. Based on the application of the definition, different BW types could be utilized.

**Electronic supplementary material:**

The online version of this article (doi:10.1186/1471-2369-15-176) contains supplementary material, which is available to authorized users.

## Background

Acute kidney injury (AKI) is a frequent clinical syndrome among hospitalized, and particularly in critically ill, patients. The incidence of AKI occurring in patients admitted to intensive care units (ICUs) ranges from 30-60% [1]. Independently associated with both short and long-term mortality [2–4], AKI-associated mortality was reported to be as high as 23% [5].

Previously, there have been many different definitions of AKI used in the literature and clinical practice. Various classifications, RIFLE (Risk, Injury, Failure, Loss of function, and End-stage renal disease [ESRD]) criteria in 2004, AKIN (Acute Kidney Injury Network) criteria in 2007, and most recently, KDIGO (Kidney Disease Improving Global Outcomes) criteria in 2012, have been developed and validated to standardize the diagnosis and staging the severity of AKI [6–8]. The definition of AKI is currently based on absolute or relative changes in serum creatinine (SCr) and weight-adjusted hourly urine output (UO), respectively (Table 1). A general consensus of these definitions has reduced the variation in AKI research studies findings, as the vast majority of investigations within the past decade used these similar definitions to diagnose and stage AKI.

**Table 1:** **KDIGO criterion for diagnosis and staging of AKI** [8]

Stage	Serum creatinine	Urine output
1	1.5-1.9 times baseline OR 0.3 mg/dl increase	< 0.5 ml/kg/h for 6-12 hours
2	2.0-2.9 times baseline	< 0.5 ml/kg/h for ≥12 hours
3	3.0 times baseline OR Increase in serum creatinine to ≥4.0 mg/dl OR initiation of replacement therapy	< 0.3 ml/kg/h for ≥24 hours OR Anuria for ≥12 hours

Studies have found oliguria can be an early indicator of kidney dysfunction and is independently associated with poor mortality and morbidity [9, 10]. Body weight (BW) is an important factor and used when normalizing the UO for weight and time. The UO thresholds that are currently normalized to BW may need to be adjusted to an ideal body weight (IBW) to account for obesity or cachexia. However, It is not clearly specified whether actual body weight (ABW) or IBW, should be used to define AKI. Although a large number of recent studies reported UO criterion, the majority of them do not specify which body weight was used, leading to heterogeneity among different studies [8, 11, 12]. In this report, we showed using different methods of measuring or calculating body weight can impact the sensitivity and specificity of the AKI definition.

This study aims to (1) evaluate and compare the incidence of AKI and its staging according to UO criteria, using ABW versus IBW and (2) investigate how using different BW calculations would affect predictive performance of AKI on 90-day mortality and other morbidities in critically ill patients.

## Methods

### Subjects and methods

This was a single-center retrospective study conducted at a tertiary referral hospital. We studied all adult patients (age ≥18 years) admitted to an intensive care unit (ICU) in our hospital (Mayo Clinic Hospital – Rochester, Rochester, MN) for a minimum of six continuous hours from January to February 2010. We excluded patients without indwelling urinary catheters, patients with a history of ESRD, patients who received any dialysis modalities within 14 days prior to the ICU admission, and patients who did not provide research authorization. This study was approved by the Mayo Clinic Institution Review Board with a waiver of patient consent because of retrospective and non-interventional fashion of this study.

### AKI diagnosis and staging

In the electronic medical record (EMR), AKI was diagnosed and staged based solely on the UO criterion of the KDIGO definition (Table 1) [13]. Hourly UO data in the ICU EMR was manually reviewed by a trained critical care physician (AA), blinded to patients’ vital status. Patient weight is measured and recorded on daily basis, using digital weighting scale. Height is also measured by measurement tape, at least one time at ICU admission. The documented BW (in kg) on the first day of ICU admission was used as the ABW. The IBW was calculated using the following equations [14]:


For patients who had an ABW greater than 1.3 times their IBW, the IBW was further adjusted using the following formula [15]:


Based on the above calculations, the initial reviewer determined a disagreement of AKI stages in 86 patients. The EMRs of these 86 patients were subsequently reviewed by a second physician (CT) who was blinded to the result of the first reviewer. Disagreements of AKI stages between the two reviewers were adjudicated by a joint review between these two physicians.

In a sensitivity analysis, we used both SCr and UO definitions for diagnosis and staging of AKI (Table 1). AKI stage was assigned according to the highest stage using either the SCr or UO criterion. The baseline SCr was defined as the mean value of all SCr values measured within 1 year before hospital admission. In 38 (7.7%) patients whose measured SCr was not available, the baseline SCr was estimated by the Modification of Diet in Renal Disease (MDRD) equation, assuming baseline estimated glomerular filtration rate (eGFR) of 75 ml/min per 1.73 m^2^ [16].

### Clinical outcomes

The primary outcome was 90-day mortality following the index ICU admission. Vital statistics were first obtained by reviewing the patients’ registration and EMRs to identify patients’ mortality status beyond 90 days after ICU admission. In patients whose vital status at 90 days after ICU admission was unknown, due to lack of follow-up (4.8%), the Social Security Death Index was used [17].

### Statistical analysis

All continuous variables were reported as medians with interquartile ranges (IQR). All categorical variables were reported as counts with percentages. In the event of missing information, data were not muted. For patients with multiple ICU admissions, only the first ICU admission during the study period was included in analysis. The difference in the ABW- versus IBW-based AKI diagnosis was assessed using a McNemar’s test. The difference in the time-to-AKI diagnosis based on ABW and IBW was assessed using a paired t test. The agreement of AKI diagnosis and staging based on ABW and IBW was assessed using Cohen’s weighted kappa coefficient with linear weight between AKI stages. We adjusted odd ratios (ORs) for pre-specified variables including, age and APACHE III score, to assess 90-day mortality among patients who met the UO criterion, regardless of type of BW measured, and those who met UO criterion only (according to either ABW or IBW but not the other) compared to non-AKI patients. The association between AKI stages and 90-day mortality was assessed using a logistic regression analysis. The predictive performance of the UO criterion, using different BW calculations, for 90-day mortality was assessed by c-statistics; after which we compared their performances using Delong’s test. A two-sided *P* value of < .05 was considered statistically significant. Sensitivity and specificity of ABW-based and IBW-based AKI diagnosis were calculated using serum creatinine-based definition as the reference. All analyses were performed using JMP statistical software (version 9.0, SAS, Cary, NC).

## Results

During the study period, 639 critically ill patients were admitted to ICU. Of these, 146 were excluded: 31 had ESRD or received dialysis within 14 days prior to ICU admission, 101 had no indwelling urinary catheter for hourly UO monitoring, and 14 had an ICU length of stay of <6 hours. A total of 493 patients were analyzed. The clinical characteristics of these patients at the ICU admissions and their outcomes are summarized in Table 2. The median age was 67 years (IQR 54-77); 54% were men and 30% had chronic kidney disease. The median body mass index (BMI) was 28 kg/m^2^ (IQR 24-33). The median ABW and IBW were 82 (IQR 68-96) and 70 (IQR 60-77) kg respectively (p <0.001).

**Table 2:** **Clinical characteristics and outcomes of critically ill patients admitted in ICU during the study period**

Characteristics	Total (n = 493)
Age, year, median (IQR)	67 (54-77)
Male sex, n (%)	264 (54)
White, n (%)	440 (89)
BMI, kg/m^2^, median (IQR)	28 (24-33)
Body weight, kg, median (IQR)	
- Actual body weight	82 (68-96)
- Ideal body weight	70 (60-77)
Baseline creatinine, mg/dL, median (IQR)	1 (0.8-1.2)
Comorbidities	
- DM	115 (23)
- Coronary artery disease	59 (12)
- Stroke	49 (10)
- Congestive heart failure	34 (7)
- Chronic pulmonary disease	119 (24)
- Cirrhosis	27 (5)
- Chronic kidney disease*, n (%)	148 (30)
ICU type	
- Medical ICU	216 (44)
- Surgical ICU	173 (35)
- Mixed ICU	104 (21)
APACHE III score, median (IQR)	44 (32-59)
SOFA score, median (IQR)	4 (2-7)
ICU length of stay, hour, median (IQR)	28 (20-55)
90-day mortality, n (%)	79 (16)

*Chronic kidney disease was defined by K/DOQI CKD stage III or worse (estimated glomerular filtration rate <60 ml/min/1.73 m^2^ according to MDRD formula).

*Abbreviation:* *APACHE* Acute Physiology and Chronic Health Evaluation, *SOFA* Sequential Organ Failure Assessment, *BMI* body mass index.

### AKI diagnosis and staging using ABW and IBW

When patients’ ABW measurements were used, AKI was diagnosed in 204 (41.4%) of the patients, with 21.5% in stage 1, 15.4% in stage 2 and 4.5% in stage 3. Using IBW, AKI occurred in 154 (31.2%) patients with 18.7% in stage 1, 9.1% in stage 2 and 3.4% in stage 3. Accordingly, using ABW could identify more AKI cases than IBW (*P* < .001) (Table 3).

**Table 3:** **AKI diagnoses and staging using UO criterion with ABW and IBW**

AKI stage (Actual BW)	AKI stage (ideal BW)				Total N (%)
	0	1	2	3	
0	289 (58.6)	0 (0)	0 (0)	0 (0)	289 (58.6)
1	45 (9.1)	61 (12.4)	0 (0)	0 (0)	106 (21.5)
2	5 (1.0)	31 (6.3)	40 (8.1)	0 (0)	76 (15.4)
3	0 (0)	0 (0)	5 (1.0)	17 (3.4)	22 (4.5)
Total, N (%)	339 (68.8)	92 (18.7)	45 (9.1)	17 (3.4)	493

Kappa = 0.78 (95% CI 0.73-0.84) and percentage agreement = 89.9% for AKI diagnosis.

Kappa = 0.77 (95% CI 0.73-0.82) and percentage agreement = 82.6% for AKI staging.

*Abbreviation:* *AKI* acute kidney injury, *BW* body weight, *CI* confidence interval.

The percentage agreement for AKI diagnosis, using the two different body weight assertation methods, was 89.9% with a kappa of 0.78 (95% CI, 0.73-0.84). Results show that ABW and IBW both agreed in 154 AKI cases and 289 non-AKI cases. Using a different BW measurement resulted in a discrepancy in AKI diagnosis in 50 cases (10.1%). All of these 50 patients had AKI according to ABW but not IBW. The number of patients who had AKI with IBW but not with ABW was zero. The percentage agreement for AKI staging was 82.6% with a kappa of 0.77 (95% CI, 0.73-0.82).

### Time-to-AKI diagnosis using ABW and IBW

We found that in patients who were diagnosed with AKI, regardless of body weight methodology. Using ABW detected AKI significantly earlier than using IBW with the mean difference in time-to-diagnosis of 4.0 hours (95% CI, 0.2-7.7; *P* = .04). The ABW method was able to identify AKI earlier than IBW in 20.1% of these patients, whereas 79.2% had AKI at the same time, according to both types of BW.

### Risk for 90-day mortality

**Figure 1:**
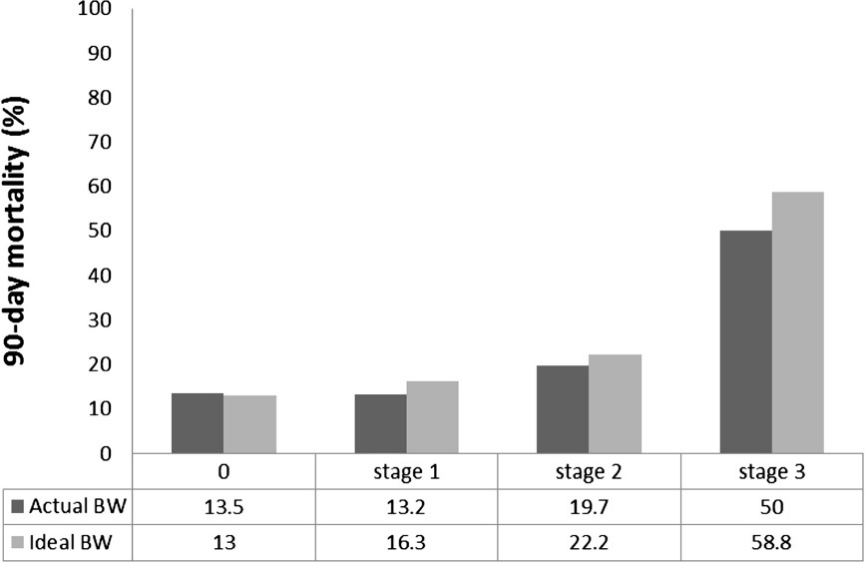
**90-day mortality rate according to AKI stages (UO definition).**

Of these patients, 16% (n = 79) died within 90 days after their ICU admission. The 90-day mortality rates after ICU admission for AKI stages by ABW and IBW are shown in Figure 1. With ABW, there was a statistically non-significant trend toward a higher 90-mortality rate in AKI cases compared to non-AKI cases (19.6% vs. 13.5%; *P* = .06). In contrast, with IBW, the 90-day mortality rate was significantly higher in AKI cases compared to non-AKI cases (22.7% vs. 13.0%; *P* = .006).

Compared to patients who did not have AKI, patients who had AKI, regardless of the BW calculation, had an adjusted OR for 90-day mortality of 1.76 (95% CI, 1.05-2.95), whereas patients who had AKI according to ABW but not by IBW had non-significant increase in 90-day mortality risk (adjusted OR = 0.76; 95% CI, 0.25-1.91) (Table 4). Calculating the performance for the prediction of 90-day mortality, the C-statistic for ABW and IBW methods were 0.58 and 0.60, respectively. C-statistics were not statistically different between the two methods (*P* = .37).

**Table 4:** **90**-**day mortality risk**

Actual BW	Ideal BW	N	90-mortality rate	Adjusted OR (95% CI)*
AKI	AKI	154	22.7%	1.76 (1.05-2.95)
AKI	No AKI	50	10%	0.76 (0.25-1.91)
No AKI	AKI	0	n/a	n/a
No AKI	No AKI	289	13.5%	Reference

*OR is adjusted for age and APACHE score.

### Sensitivity analysis

When both SCr and UO criteria were used for AKI diagnosis and staging, similarly, using ABW could detect more AKI cases (50.5% vs. 42.4%; *P* < .001) (see Additional file 1: Table S1, Table S2, Table S3, Figure S1). In term of outcome prediction, compared to non-AKI cases, the mortality was significantly higher in AKI cases defined by ABW (19.3% vs. 12.7%; *P* = .04) as well as by IBW (21.1% vs. 12.3%; *P* = .01) in comparison with non-AKI patients. There was no significant difference in c-statistic for 90-day mortality discrimination between ABW and IBW (0.60 vs. 0.59, *P* = .33).

Using SCr criteria as reference standard for AKI diagnosis, the sensitivity of ABW-based and IBW-based AKI diagnosis were 64.8% and 57.0% respectively (*P* = .002). The specificity of ABW-based and IBW-based AKI diagnosis were 66.4% and 77.6% respectively (*P* < .001). The positive predictive values for ABW-based and IBW-based AKI diagnosis were 41.5% and 48.3% respectively (*P* = .20). The negative predictive value for ABW-based and IBW-based AKI diagnosis were 83.7% and 83.1% respectively (*P* = .84).

## Discussion

We conducted a large retrospective cohort study to examine the effect of using different body weight methods, ABW and IBW, on epidemiology and prognostication performance of AKI definition. This study showed that using ABW for AKI diagnosis not only can identify more patients with AKI but also identifies these cases earlier. Although, specificity may decrease due to false positive cases, as some AKI cases had a similar prognosis as non-AKI cases. Normalization of UO with different body weight will significantly affect the incidence of AKI in ICUs, leading to heterogeneity among different studies. The types of body weight used to normalize UO need to be clearly stated as the UO criterion is defined for both AKI diagnosis and classification.

Up to two-thirds of patients in the ICU are reported as having AKI [18]. Even a modest degree of renal insufficiency in the ICU is associated with increased in-hospital mortality [19, 20]. Prevention and early diagnosis of AKI are the keys to minimizing further insults. While awaiting for the ongoing investigations for novel biomarkers for the early AKI detection [21], using ABW for the UO criterion will provide better sensitivity and earlier time-to-AKI diagnosis in clinical practice in order to promptly detect AKI and prevent further kidney damage.

Recently, there have been studies proposing that the current UO definition may be too liberal and may over-diagnose AKI in critically ill patients [11, 12]. With the current definition, using IBW makes the AKI definition more specific. Most of patients who were diagnosed with AKI, according to ABW but not according to IBW, had temporary borderline oliguria and the outcome of patients in this group was similar to patients without AKI.

A counterintuitive finding in this study was that the 90-day mortality rate among patients who had AKI according to ABW but not IBW was statistically non-significant lower than patients who had no AKI (10% vs 13.5%; p = 0.50). One possible explanation for the observed trend, is obesity paradox phenomenon as the median BMI of the first group was significantly higher than of the latter group [32 (IQR 29-35) vs 27 (23-32) kg/m^2^; *P* < .001]. A few epidemiological studies have recently reported improved outcomes in obese patients in ICU when compared with those with normal weight [22]. Druml *et. al*. studied the impact of body mass on incidence and prognosis of AKI in the ICU and demonstrated a greater survival benefit after AKI requiring renal replacement therapy in obese patients compared to underweight or normal weight patients [23]. When we added BMI into the 90-day mortality prediction model, it showed a trend toward increased adjusted OR of 90-day mortality from 0.76 (0.25-1.91) to 0.95 (0.30-2.42).

The choice of using ABW or IBW for AKI diagnosis and classification depends on the purpose of the AKI definition. In clinical practice, AKI prevention and early treatment may improve patient outcomes. Therefore, for screening purposes in clinical practice, we support the use of ABW to normalize UO for AKI diagnosis, as it can potentially identify more AKI cases earlier. On the other hand, for research studies that enroll patients with AKI for invasive medical intervention, using IBW may be more appropriate, as it is likely to select patients who are going to benefit the intervention.

Our study has some limitations. This report is a retrospective study and inherently subjective to biases of a retrospective study. The other potential limitation is the small number of underweight patients admitted to our ICU. In the majority of our patients ABW was more than their IBW. This made it difficult to generalize our study findings to patient populations with a lower BMI. In our cohort, 38 patients had a BMI of ≤20 kg/m^2^. Among these underweight patients, AKI was diagnosed in 10 (26.3%) using ABW and in 9 (23.7%) using IBW (*P* = .32). This suggests that the need for BW adjustment for AKI diagnosis when using UO criterion might not be necessary in patients with a low BMI. However, further studies are needed to confirm the diagnostic and predictive abilities of different BW calculation for underweight patients. Second, we did not have information regarding fluid balance as the fluid administration before ICU admission may affect the actual BW measured at ICU admission. However, we selected the first body weight measured in ICU to minimize the impact of fluid balance on actual body weight.

Yet, with these limitations our study carries some strength as well. It is a large cohort of patients in medical and surgical ICUs with different critical illnesses. We were able to access hourly UOt in this large sample. In addition, this is the only report that focuses on the type of BW for normalization of UO criterion on AKI definition.

## Conclusions

In summary, using ABW for UO criterion increases its sensitivity and allows earlier diagnosis of AKI; however it does not add to the prediction performance of the AKI definition for 90-day mortality when compared with IBW. Based on our data, we suggest ABW to be called “sensitivity BW” for risk stratification purposes, whereas IBW should be called “specificity BW” for enrollment in more invasive diagnostic and therapeutic measures.

## Electronic supplementary material

Additional file 1: Table S1: Baseline characteristics of ICU patients grouped by the occurrence of AKI using ABW and IBW for diagnosis. **Table S2.** AKI diagnoses and staging according to SCr and UO definition using actual and ideal BW. **Table S3.** 90-day mortality risk. **Figure S1.** 90-day mortality rate according to AKI stages (SCr and UO definition). (PDF 189 KB)

## Abbreviations

ABW: Actual body weight
AKI: Acute kidney injury
BW: Body weight
CI: Confidence interval
IBW: Ideal body weight
ICU: Intensive care unit
IQR: Interquartile range
UO: Urine output
SCr: Serum creatinine.
