# Supplementary material for: Actual versus ideal body weight for acute kidney injury diagnosis and classification in critically Ill patients
Source: BMC Nephrol. 2014 Nov 15;15:176. doi: 10.1186/1471-2369-15-176 (PMC4236495; doi:10.1186/1471-2369-15-176)

Table S1 Baseline characteristics of ICU patients grouped by the occurrence of AKI using ABW and IBW for diagnosis

Characteristic	ABW: AKI<br>IBW: AKI	ABW: AKI<br>IBW: No AKI	ABW: No AKI<br>IBW: No AKI	p
Age, year	70 (58-77)	68 (57-75)	66 (52-77)	0.12
Male sex	80 (52)	20 (40)	164 (57)	0.08
White	138 (90)	45 (90)	257 (89)	0.96
BMI, kg/m^2	28 (24-34)	32 (29-35)	27 (23-32)	<0.001
Body weight, kg				
- Actual BW	82 (70-98)	89 (78-103)	81 (65-94)	0.004
- Ideal BW	70 (60-78)	70 (61-78)	69 (58-76)	0.23
Baseline creatinine, mg/dL	1.1 (0.8-1.4)	0.9 (0.8-1.2)	1 (0.8-1.2)	0.15
Chronic kidney disease	59 (38)	12 (24)	77 (27)	0.02
ICU type				0.46
- Medical ICU	75 (49)	21 (42)	120 (42)	
- Surgical ICU	48 (31)	8 (16)	104 (36)	
- Mixed ICU	31 (20)	21 (42)	65 (22)	
APACHE III score	47 (36-61)	42 (30-53)	44 (32-59)	0.08

Continuous data are presented as median (IQR), Categorical data are presented as n (%).

Table S2: AKI diagnoses and staging according to SCr and UO definition using actual and ideal BW

AKI stage (Actual BW + SCr)	AKI stage (ideal BW + SCr)				Total N (%)
	0	1	2	3	
0	244 (49.5)	0 (0)	0 (0)	0 (0)	244 (49.5)
1	36 (7.3)	92 (18.7)	0 (0)	0 (0)	128 (26.0)
2	4 (0.8)	26 (5.3)	50 (10.1)	0 (0)	80 (16.2)
3	0 (0)	0 (0)	1 (0.2)	40 (8.1)	41 (8.3)
Total, N (%)	284 (57.6)	118 (23.9)	51 (10.3)	40 (8.1)	493

Kappa = 0.84 (95% CI 0.79-0.89) and percentage agreement = 92 %
Kappa = 0.86 (95% CI 0.82-0.89) and percentage agreement = 86.4 %

Table S3: 90-day mortality risk

Actual BW	Ideal BW	N	90-mortality rate	Adjusted OR(95% CI)*
AKI	AKI	209	21.1%	1.65 (0.99-2.77)
AKI	No AKI	40	10.0%	0.83 (0.23-2.32)
No AKI	No AKI	244	12.7%	Reference

* OR is adjusted for age and APACHE score

Figure S1: 90-day mortality rate according to AKI stages (SCr and UO definition)

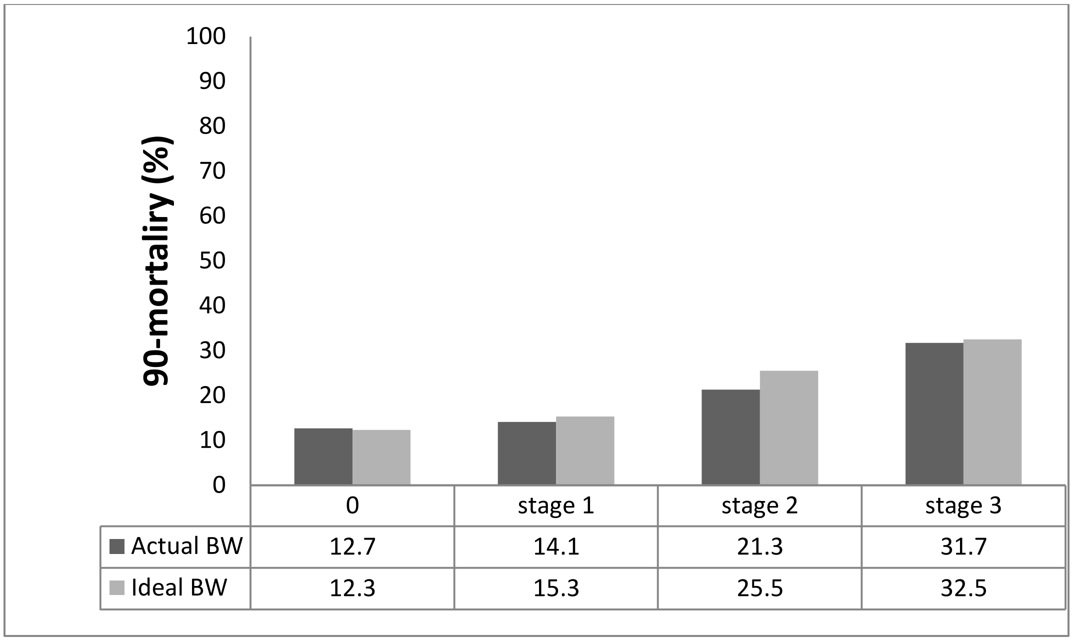

Supplement: Supplementary file 1 — Additional file 1: Table S1: Baseline characteristics of ICU patients grouped by the occurrence of AKI using ABW and IBW for diagnosis. Table S2. AKI diagnoses and staging according to SCr and UO definition using actual and ideal BW. Table S3. 90-day mortality risk. Figure S1. 90-day mortality rate according to AKI stages (SCr and UO definition). (PDF 189 KB) [file 12882_2014_865_MOESM1_ESM.pdf]
